# A case of welder's pneumoconiosis treated with corticosteroid followed by nintedanib

**DOI:** 10.1016/j.rmcr.2022.101729

**Published:** 2022-08-19

**Authors:** Hideyuki Kaida, Takuhide Utsunomiya, Yohei Koide, Yusuke Ueda, Kenji Wada, Yuji Yoshida, Yoshiaki Kinoshita, Hisako Kushima, Hiroshi Ishii

**Affiliations:** a Department of Respiratory Medicine, Miyakonojo Medical Association Hospital, Miyazaki, Japan; b Department of Respiratory Medicine, Fukuoka University Chikushi Hospital, Fukuoka, Japan

**Keywords:** Welder's pneumoconiosis, Alveolar hemorrhage, Progressive fibrosing interstitial lung disease, Nintedanib, CT, computed tomography, PF-ILD, progressive fibrosing interstitial lung disease, IPF, idiopathic pulmonary fibrosis

## Abstract

A 32-year-old man who had worked as a welder for 13 years was hospitalized for a fever and hemosputum with dyspnea. He was diagnosed with welding fume-associated lung disease with alveolar hemorrhaging and acute respiratory failure. Despite surviving the acute phase with corticosteroid therapy, hypoxemia persisted after a month and a half, requiring home oxygen therapy. As a result of the introduction of nintedanib, his clinical findings gradually improved, and the patient was weaned from oxygen therapy after six months. Inhalation of a large amount of welding fumes in a short period can cause alveolar hemorrhaging and prolonged pulmonary dysfunction.

## 1 Introduction

Welding fume-associated lung disease was once recognized as a benign pneumoconiosis called Welder's siderosis, histologically showing deposits of iron oxide without pulmonary fibrosis [1], but it has recently been believed that pulmonary fibrosis occurs when large amounts of iron oxide in welding fumes accumulate in the lungs [[2], [3], [4]].

We herein report a case of acute respiratory failure with alveolar hemorrhaging after inhalation of a large amount of welding fumes that was treated with corticosteroids followed by an antifibrotic agent to address the prolonged decline in the respiratory function.

## 2 Case report

**Fig. 1:**
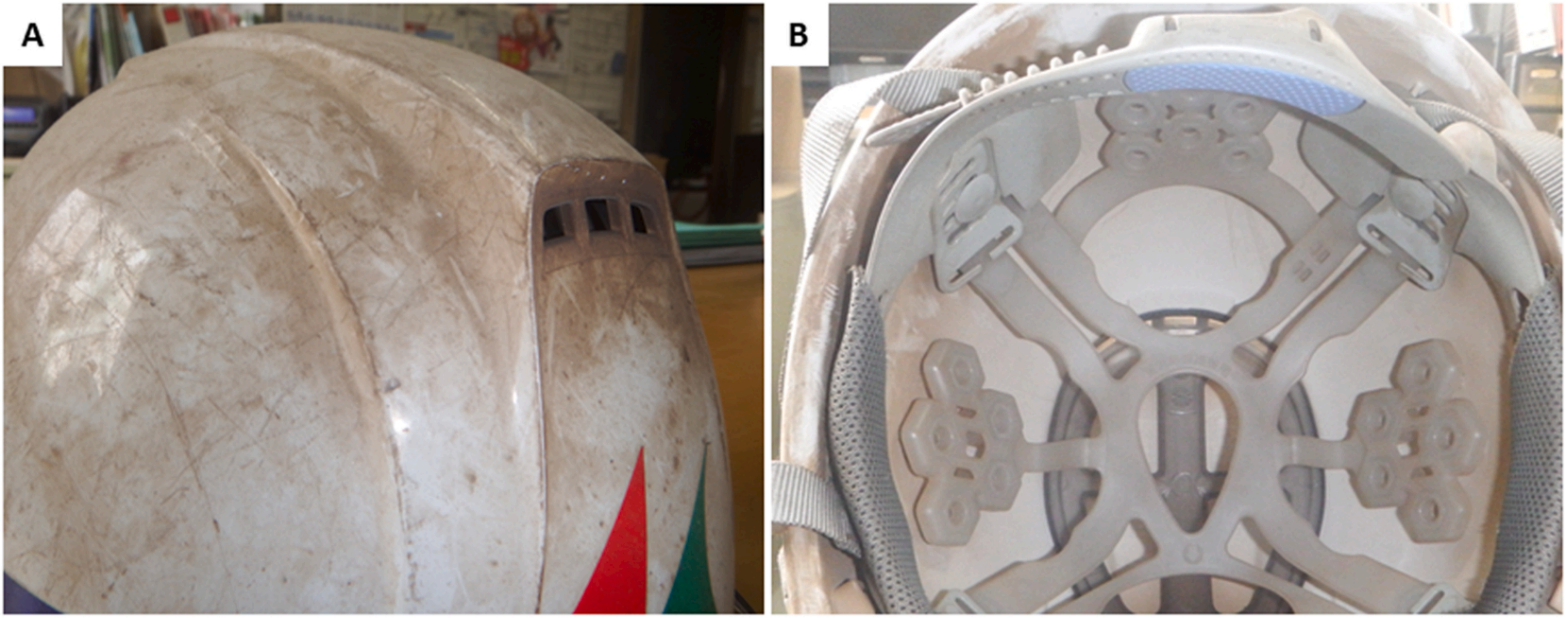
The patient's helmet, showing a lot of brown dust both outside (A) and inside (B). (For interpretation of the references to colour in this figure legend, the reader is referred to the Web version of this article.)

A 32-year-old man who had worked as a welder for 13 years with no remarkable medical history was referred to the emergency room for a fever and hemosputum with dyspnea (modified Medical Research Council breathlessness scale-4). He was a current smoker of 16.5 pack-years. He had been properly using a dust-proof mask while working, but a large amount of welding fumes had been generated two weeks prior to the visit, clogging the dust filter after half a day and causing the patient's helmet to turn brown (Fig. 1). No one, including the Labor Standards Inspection Office staff, could explain what caused the large amount of fumes. His fever and cough appeared three days before admission, and the hemosputum and dyspnea appeared the day before.

**Fig. 2:**
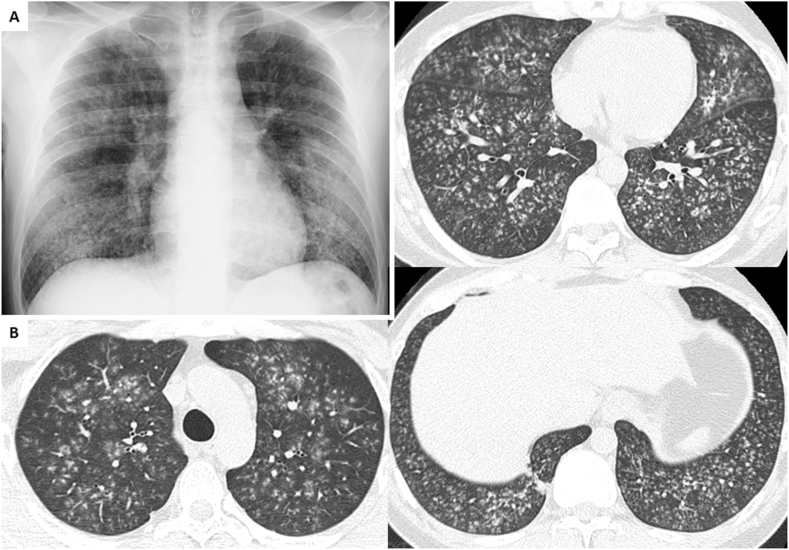
Chest radiography on admission, showing diffuse reticulonodular abnormalities in both lung fields (A). Chest computed tomography on admission showing multiple centrilobular small nodules and ground-glass appearances in both lungs (B).

**Fig. 3:**
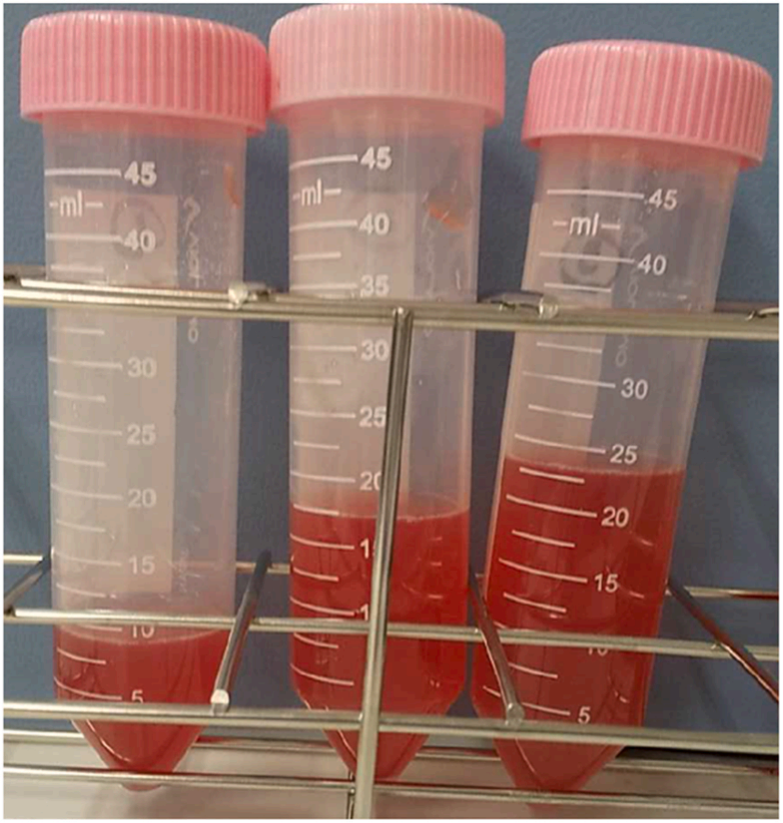
Bloody fluid recovered by bronchoalveolar lavage.

On admission, his body temperature was 38.3 °C, respiratory rate 27/min, blood pressure 117/74 mmHg, and pulse rate 94/min. No abnormal breath sounds were heard. The laboratory data showed white blood cell counts of 21,000/μL (neutrophils 81.8%, lymphocytes 6.4%, monocytes 11.2%, eosinophils 0.2%, basophils 0.4%), hemoglobin 13.4 g/dL, platelets 438,000/μL, lactate dehydrogenase 261 U/L (normal value < 222 U/L), C-reactive protein 40.7 mg/dL, ferritin 1702 ng/mL (<200 ng/mL), D-dimer 2.5 μg/mL (<1.0 μg/mL), Krebs von den Lungen-6184 U/mL (<500 U/mL), surfactant protein-D 408.7 ng/mL (<110.0 ng/mL), and negative findings for anti-nuclear antibody, myeloperoxidase-anti-neutrophil cytoplasmic antibody and anti-glomerular basement membrane antibody. An arterial blood gas analysis revealed hypoxemia with pH 7.476, PaO_2_ 83.1 Torr, and PaCO_2_ 36.2 Torr under 5 L of oxygen inhalation. Chest radiography and computed tomography (CT) showed diffuse small nodules and ground-glass opacities in both lungs (Fig. 2), and bronchoalveolar lavage revealed bloody fluid (Fig. 3) with an abundance of hemosiderin-laden alveolar macrophages.

**Fig. 4:**
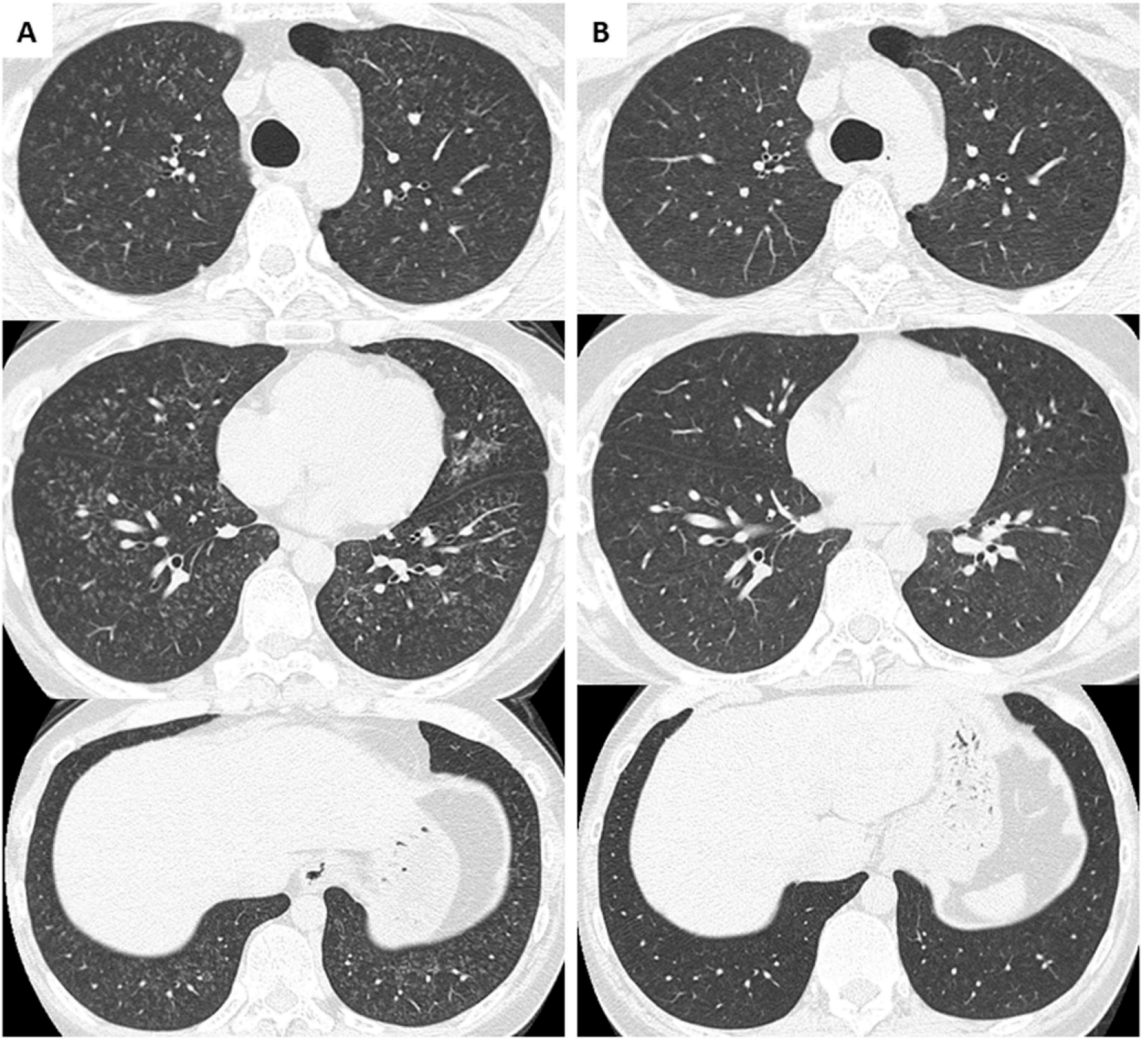
Chest computed tomography showing improvement on day 44 (A) and further improvement on day 460 (B).

**Fig. 5:**
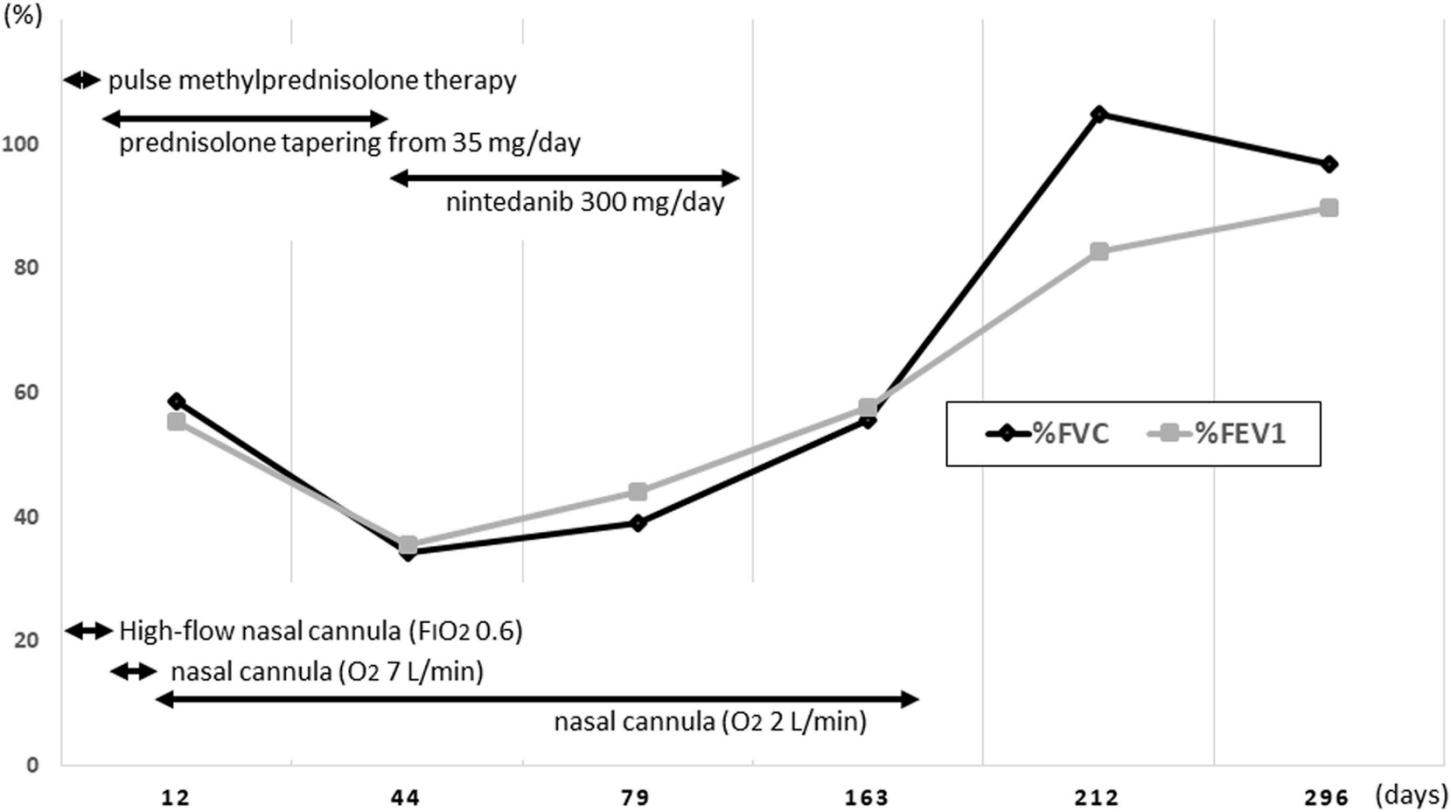
A clinical course of the patient. %FVC; forced vital capacity, % predicted, %FEV1; forced expiratory volume in 1 sec, % predicted.

Since there were no physical or serological findings suggestive of vasculitis or connective tissue diseases, the patient was diagnosed with welding fume-associated lung disease with alveolar hemorrhaging and acute respiratory failure. He survived the acute phase with corticosteroid therapy (pulse methylprednisolone therapy [1000 mg for 3 days] followed by prednisolone tapering [35 mg/day and tapered by 5 mg every week]). However, despite improvement in his chest CT findings (Fig. 4A), he remained smoke-free but still had persistent hypoxemia and a decreased respiratory function after a month and a half, requiring home oxygen therapy to manage (Fig. 5). As a result of the introduction of nintedanib treatment 44 days after hospitalization, his clinical findings gradually improved within the subsequent two months. Although nintedanib treatment was discontinued due to liver dysfunction two months after the initiation, the patient was eventually weaned from home oxygen therapy after a total of six months (Fig. 5). He did not engage in any further welding work, and his respiratory function normalized after seven months and did not deteriorate thereafter. CT more than a year later showed further improvement to simply pale micronodules (Fig. 4B).

## 3 Discussion

Welder's pneumoconiosis/welder's lung, which is caused by the inhalation of welding fumes, is a major pneumoconiosis in Japan. The major component of welding fumes is iron oxide [5], and metal-rich particles are deposited and retained in the lower respiratory tract [6]. Although welder's pneumoconiosis was initially considered to be inert without evidence of accompanying pulmonary fibrosis, it has been noted to cause pulmonary fibrosis in some cases [3,7].

On chest images, typical welder's pneumoconiosis shows small centrilobular nodules in both lungs, and some cases show mild fibrotic changes in subpleural areas of the lower lung lobes [4]. In the present case, diffuse small centrilobular nodules were observed on CT in the acute phase, which was consistent with previous reports. In addition, there were ground-glass appearances bilaterally, probably due to diffuse alveolar hemorrhaging. However, the CT findings during the period of prolonged hypoxemia and respiratory dysfunction were residual diffuse centrilobular nodules, rather than fibrotic changes in subpleural areas of the lower lung lobes. The patient showed not only prolonged restrictive ventilation impairment but also prolonged obstructive ventilation impairment (Fig. 5), which might have been caused by airway-centered fibrosis [8] as well as latent perilobular fibrosis due to inhalation of welding fumes, although histopathological studies were not performed.

Burke et al. [3] reported a series of welder's pneumoconiosis cases that were heavily exposed to welding fumes in a poorly ventilated workplace for many years. These patients developed respiratory dysfunction and hypoxemia on exertion, and a histological evaluation of their lungs showed partial fibrosis of the lung interstitium. Particulate material of welding fume was found in coincidence with the areas of fibrosis, and a mineral analysis showed a pronounced peak in iron. The overload phenomenon, in which large amounts of iron oxide in welding fumes accumulate in the lungs and can cause pulmonary fibrosis, has been proposed as a potential mechanism [5]. This means that, in cases of mild exposure to small amounts of welding fumes, iron oxide and hemosiderin-laden macrophages are present in the bronchioles and surrounding alveolar space, and their numbers are reduced by muco-ciliary movement, resulting in clinical improvement. However, in cases of exposure to large amounts of fumes, the aggregation of iron oxide and hemosiderin-laden macrophages may exceed the processing capacity of muco-ciliary movement. Muller and Verhoff [7] reviewed 38 biopsied or autopsied lung tissues and proposed the histological grading of sideropneumoconiosies based on the degree of fibrosis. Their proposal might well reflect the degree of the above-mentioned overload phenomenon.

Recently, progressive fibrosing interstitial lung disease (PF-ILD) has been redefined as a new clinical syndrome with similar genetics, pathophysiology, and a natural history to idiopathic pulmonary fibrosis (IPF) [9]. Novel uses of antifibrotic therapy are emerging due to a paucity of evidence-based treatments for multiple ILD subtypes. An annual decline 5%–10% of the forced vital capacity was associated with early death in PF-ILD patients, irrespective of the underlying ILD diagnosis and fibrotic pattern on chest CT, just as for untreated IPF patients [10]. Although it is somewhat difficult to determine whether the present case corresponds to PF-ILD, treatment with antifibrotic agent was introduced because of the patient's relatively young age and the prolonged hypoxemia and restrictive ventilation impairment despite the improvement in imaging findings after steroid therapy. As the patient's liver function normalized upon discontinuation of nintedanib, he was considered to have drug-induced liver injury. Although exposure to high concentrations of fumes can cause severe hepatic iron overload [5], the patient's serum ferritin had already normalized at the start of nintedanib treatment.

Welding fumes, especially metal arc welding, have been reported to increase the toxic lung response by enhancing the production of highly reactive oxygen radicals and inflammatory cytokines by macrophages [11]. Although alveolar hemorrhaging due to inhalation of welding fumes is quite rare, previous reports [12,13] and the present case have demonstrated that welding fumes can cause airway damage and inflammation, injuring the bronchial and alveolar epithelium and vasculature and leading to acute respiratory failure with hemorrhaging.

In summary, this case report highlights the fact that inhalation of a large amount of welding fumes in a short period can cause acute respiratory failure accompanied by alveolar hemorrhaging. Although there is a possibility of a spontaneous improvement after corticosteroid therapy in the present case, a prolonged pulmonary dysfunction in welder's lung might be a target for treatment with antifibrotic agents.

## Declaration of competing interest

The authors have no conflicts of interest directly relevant to the content of this article.
